# A critical review of front‐loading: A maladaptive drinking pattern driven by alcohol's rewarding effects

**DOI:** 10.1111/acer.14924

**Published:** 2022-10-14

**Authors:** Cherish E. Ardinger, Christopher C. Lapish, Cristine L. Czachowski, Nicholas J. Grahame

**Affiliations:** ^1^ Addiction Neuroscience, Department of Psychology and Indiana Alcohol Research Center Indiana University – Purdue University Indianapolis Indianapolis Indiana USA; ^2^ Stark Neuroscience Research Institute Indiana University – Purdue University Indianapolis Indianapolis Indiana USA

**Keywords:** alcohol, alcohol use disorder, drinking patterns, front‐loading, high‐intensity drinking, motivation, tolerance

## Abstract

Front‐loading is a drinking pattern in which alcohol intake is skewed toward the onset of reward access. This phenomenon has been reported across several different alcohol self‐administration protocols in a wide variety of species, including humans. The hypothesis of the current review is that front‐loading emerges in response to the rewarding effects of alcohol and can be used to measure the motivation to consume alcohol. Alternative or additional hypotheses that we consider and contrast with the main hypothesis are that: (1) front‐loading is directed at overcoming behavioral and/or metabolic tolerance and (2) front‐loading is driven by negative reinforcement. Evidence for each of these explanations is reviewed. We also consider how front‐loading has been evaluated statistically in previous research and make recommendations for defining this intake pattern in future studies. Because front‐loading may predict long‐term maladaptive alcohol drinking patterns leading to the development of alcohol use disorder (AUD), several future directions are proposed to elucidate the relationship between front‐loading and AUD.

## Graphical abstract

Front‐loading is a drinking pattern where intake is skewed toward the onset of reward access. This phenomenon has been reported across different alcohol self‐administration protocols using a variety of species, including humans. The hypothesis of the current review is that front‐loading emerges in response to the rewarding effects of alcohol and can be used as a measure of motivation to consume alcohol. Alternative hypotheses are assessed, and several future directions are proposed to elucidate the relationship between front‐loading and AUD.

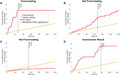

## OVERVIEW

Temporal analysis of alcohol intake patterns may lead to a more complete understanding of the development of alcohol use disorder (AUD). There is evidence that a quick rate of alcohol intake, not just a high total amount of alcohol consumed, is correlated with AUD symptoms. Recent examples supporting this include a relationship between binge‐drinking rate and AUD symptoms in adolescents (Carpenter et al., 2019), as well as heavy drinkers and individuals considered at risk for AUD self‐administering intravenous alcohol at a quicker rate than low‐risk “social drinkers” (Sloan et al., 2019). Front‐loading is a drinking pattern where intake is skewed toward the onset of reward access. Front‐loading has been reported across several different alcohol self‐administration protocols using a variety of species (Table 1). Although there is literature describing front‐loading as a measure of reward‐related behavior during the consumption of sweetened solutions, such as sucrose and saccharin (D'Aquila, 2010; Davis & Smith, 1992; Lardeux et al., 2013; Spector et al., 1998), interpretation of front‐loading in the context of alcohol intake requires careful consideration of the pharmacological effects of the drug. The hypothesis of this review is that alcohol front‐loading is driven by the rewarding effects of intoxication. This will be compared with the alternative hypotheses that front‐loading is directed at overcoming behavioral and/or metabolic tolerance, and front‐loading is driven by negative reinforcement. In addition to evaluating the hypothesis, a goal of this review is to highlight the importance of including an analysis of temporal patterns of alcohol drinking in future research. Refer Table 2 for definitions of key words used throughout this review.

**TABLE 1:** Alcohol research which reports front‐loading‐like behavior

Protocol	Species	Sex differences?	Citation	How is front‐loading calculated?
DID (20% EtOH vs. water)	B6 mice	No sex difference in front‐loading	Rhodes et al. (2007)	Assessment of sipper contacts in 30‐min bins
DID (20% EtOH vs. water)	B6 mice	N/A—males only	Linsenbardt and Boehm (2014)	Comparison to water group
DID (20% EtOH vs. water)	B6 mice	N/A—males only	Wilcox et al. (2014)	Intake in the early part of the session is compared with a later part of the session
DID (20% EtOH)	B6 mice	F w/ EtOH DID history front‐load more quinine‐adulterated alcohol than M	Bauer et al. (2021)	30 min EtOH intake of EtOH‐history group compared with water‐history group
DID (20% EtOH vs. water)	B6 mice	N/A—males only	Salling et al. (2018)	Comparison to water group
DID (20% EtOH vs. water)	HAP1 mice	No interaction of sex. All graphs shown collapsed on sex	Linsenbardt and Boehm (2015)	Comparison to water group
DID (20% EtOH vs. water)	HAP2;3 mice	No interaction of sex. All graphs shown collapsed on sex	Ardinger et al. (2020)	Comparison to water group; comparison to flat distribution of intake
DID (20% EtOH vs. water)	HDID1, HDID2, and Hs‐Npt (progenitor) mice	No sex difference in front‐loading	Jensen et al. (2021)	Comparison of time to reach >80 mg/dl BEC (HDID1/2 vs. Hs‐Npt)
IA2BC (24‐h MWF, 20% EtOH and water)	Wistar rats	N/A—m2B ales only	Darevsky et al. (2019)	Comparison to flat distribution of intake
Operant self‐administration (FR1, FR3)	Wistar rats	F front‐load more than M in 30 min FR1 session; no sex differences observed in 30 or 15 min FR3 sessions	Flores‐Bonilla et al. (2021)	Intake in the early part of the session is compared with a later part of the session
Operant self‐administration (FR3)	Long‐Evans rats	N/A—males only	Jeanblanc et al. (2019)	Comparison of lever presses across different session lengths: 1 h, 30 min, and 15 min
IA2BC (24‐h MWF, 20% EtOH and water)	Sprague–Dawley rats	No sex difference in first hour intake on last 3 days	Quadir et al. (2022)	Assessment of first hour intake
IA2BC (2‐h: 15% EtOH and water) following vapor CIE or air only	B6 mice	N/A—males only	Griffin et al. (2009)	Comparison of lick pattern: CIE vs. air control
IA2BC (24‐h MWF, 20% EtOH and water)	Long‐Evans rats	N/A—males only	Carnicella et al. (2009)	Assessment of first hour intake

Abbreviations: 2BC, two‐bottle choice; B6, C57BL/6J; CIE, chronic intermittent access; DID, drinking‐in‐the‐dark; FR1/3, fixed ratio 1/3; HAP, high alcohol‐preferring; HDID, high drinking‐in‐the‐dark; IA2BC, intermittent access to 2‐bottle choice; MWF, monday, wednesday, and friday.

**TABLE 2:** Keywords used throughout the review

Key word	Definition
Front‐loading	Front‐loading is an alcohol drinking pattern where intake is skewed toward the onset of access which results in intoxication
Binge drinking	A pattern of alcohol consumption that brings blood EtOH concentration (BEC) to 0.08%—or 0.08 g of alcohol/dl—or higher in around 2 h (NIAAA, 2004)
Drinking‐in‐the‐Dark (DID)	A rodent model of binge drinking where animals receive single‐bottle access to 20% alcohol for 2 or 4 h a day, 3 h into the dark cycle, with water available the remaining 22 or 20 h (Rhodes et al., 2005)
Intermittent access two‐bottle choice (IA2BC)	A protocol where rodents are given access to alcohol (typically 10% or 20%) in one bottle and access to water in a different bottle. This is typically conducted in the home cage. The intermittency of the procedure, typically 1 day of testing on then 1 day off, has been shown to escalate alcohol drinking over sessions (Simms et al., 2008)

## STATISTICAL ASSESSMENT OF FRONT‐LOADING

Table 1 outlines a variety of ways in which front‐loading has been assessed in the alcohol field, primarily using preclinical models in which alcohol is available for a limited amount of time each day, but water is typically available all or most of the time. Many of these studies have compared alcohol drinking patterns with a water control group. Using this comparison, the alcohol group must consume more of their total session intake during an early part of the session than the water group for the pattern to be considered front‐loading. Similarly, another common way of assessing front‐loading has been to compare alcohol intake patterns to a flat distribution of intake or compare early session intake to later session intake.

One question that arises in each of these analyses is “what time period constitutes the early part of a drinking session?” This parameter is critical to the definition of front‐loading. For example, previous work featuring 2‐h alcohol access sessions have reported front‐loading as assessed within the first 10 min (Wilcox et al., 2014), 15 min (Ardinger et al., 2020, 2021; Linsenbardt & Boehm, 2014, 2015), 30 min (Bauer et al., 2021), and up to 40 min (Griffin et al., 2009) of a session. A recent study using a 4‐h “drinking‐in‐the‐dark” (DID) access protocol reports front‐loading 120 min into the session (Jensen et al., 2021). Cumulating alcohol intoxication is a key question to consider when thinking about front‐loading. It is possible that analysis with too little time at the onset of alcohol access will miss much of the dose consumed in the session and/or important front‐loading patterns; on the other hand, behavior that is assessed too late might be strongly influenced by acute pharmacological effects of alcohol that could interfere with ingestive behavior. From this perspective, intake patterns assessed an hour or more into a drinking session might be strongly affected (and potentially limited) by current intoxication levels, while assessments at, for example, 15 min would reflect behavior of animals likely not as strongly influenced by current intoxication. For these reasons, it is critical to consider pharmacokinetics of orally (or otherwise) ingested alcohol when interpreting front‐loading behavior. For preclinical models, different species (e.g., mouse vs. rat) and procedures (e.g., operant oral self‐administration reinforced with “sips” of EtOH vs. binge access to 20% EtOH using DID) would be expected to greatly affect alcohol absorption slopes. For example, DID drinking would likely yield more rapid alcohol absorption than operant oral self‐administration, which tends to prevent continuous drinking due to its response requirements. Investigators should consider these issues when determining the most appropriate temporal window for assessment of front‐loading behavior.

**FIGURE 1:**
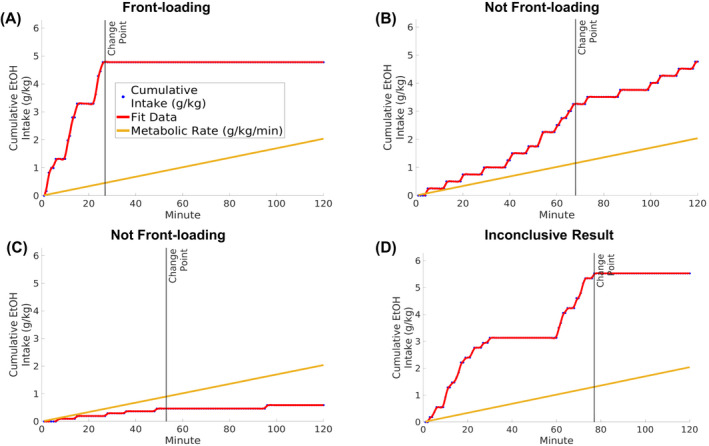
Example drinking distributions which have been categorized using the described change point analysis. (A) Front‐loading, where the drinking pattern meets all suggested criteria: (1) the strongest change point is the earliest detected change point, (2) the pre‐change point slope is significantly greater than the post‐change point slope and (3) the pre‐change point slope is greater than the rate of metabolism. (B) Not front‐loading, as the pre‐change point slope does not differ from the post‐change point slope; i.e., there is no evidence for a skew toward the onset of access. (C) Not front‐loading, as the pre‐change point slope is not greater than the rate of metabolism. (D) An inconclusive result. Inconclusive drinking patterns display a high rate of consumption at the end of the session, which is greater than any early drinking rate. However, note in this example that there is still a considerable amount of intake in the beginning of the session. Users of this analysis should consider the most clinically and experimentally relevant definition of front‐loading when determining whether front‐loading occurred. The categorizations determined by this code are only meant to serve as suggestions.

The following criteria are proposed for the analysis of alcohol front‐loading:

1. To be considered as front‐loading, subjects must display a drinking pattern which is skewed toward the onset of alcohol access in limited access situations; Figure 1A.
2. Subjects need to encounter a pharmacologically relevant dose of alcohol. If they do not, then they would not be expected to encounter alcohol's rewarding pharmacological effects that we hypothesize is driving frontloading. For this to occur, at a minimum, intake should exceed the rate of metabolism during some part of the session, which can be determined statistically through a comparison of the rate of intake to published metabolic rates of commonly used strains of mice (Grisel et al., 2002), rats (Linseman, 1989), or the model organism in question. Researchers using novel and/or transgenic strains of rats or mice should create a BEC dose‐response curve to use for this analysis. In other words, front‐loading behavior must have intoxicating consequences for the pattern of alcohol intake to be considered front‐loading. Although an intake rate slower than metabolism could still result in a skewed pattern resembling front‐loading, it would be devoid of pharmacologic consequences, and therefore of limited utility for understanding the clinical problem of intoxicating patterns of drinking in humans. Consider an individual who comes home from work and drinks a single standard‐size can of beer quickly. This would not be front‐loading if the individual stops alcohol consumption after this one beverage, similar to Figure 1C. NIAAA has set a criterion of achieving 80 mg/dl for consumption to be considered binge drinking (NIAAA, 2004), an idea that might also be considered when evaluating whether a given preclinical model makes contact with this measure of intoxicating, problem drinking. However, readers should not infer that this level must be achieved early in the session.

One way to assess if there is evidence for the presence of front‐loading as defined above is to determine if two distinct rates of consumption can be detected in the drinking session—a fast rate at the beginning of the session, followed by a slower rate that constitutes the remaining part of the drinking session. Change point analysis is a useful statistical approach that can allow for the identification of the timepoint when one transitions between rates of drinking. One caveat with this statistical approach is that some change point algorithms are biased to detect these change points in the middle of a time series. Given that front‐loading must occur in the beginning of a session, we recommend using an algorithm for detecting change points which explicitly addresses this previous limitation: the “Paired Adaptive Regressors for Cumulative Sum” (parcs) method described in Toutounji and Durstewitz (2018). Change point analysis is useful in detecting front‐loading, as it allows for an unsupervised categorization of the drinking pattern into pre‐ and post‐change point sections, which facilitates statistical analysis of skew and comparison to the metabolic rate. On our laboratory's GitHub page (https://github.com/cardinger/Detect_Frontloading), we provide simple code which uses the parcs algorithm to detect frontloading. This is accompanied with demonstrations using real data from Wistar rats, HAP2 mice, and cHAPxHDID mice. In this code, we apply three simple criteria for detecting the presence of front‐loading using this change point approach: (1) of three detected change points, the change point with the best fit statistically (as calculated using the parcs algorithm) is the earliest change point and/or is within the first half of the session. If criteria 1 is met, this best‐fit change point becomes the reference for criteria 2 and 3. If it is not met, the subject's data are categorized as inconclusive, e.g., Figure 1D. (2) The pre‐change point slope is significantly greater than the post change‐point slope, as determined through a *t*‐test comparing beta weights of pre versus post change point regressions. This assesses if there is a skew toward the beginning of the access period. (3) The pre‐change point slope exceeds the rate of alcohol metabolism. This assesses if there is evidence for intoxication. If all three criteria are met, there is strong evidence that the drinking pattern is front‐loading. See Figure 1 for examples of front‐loading and not front‐loading as categorized using this approach.

Another valid approach to assessing front‐loading is to compare the drinking pattern to a parameterized null distribution. Inherent within the definition of a skew toward the onset of access is that the drinking pattern should exceed some type of null distribution in which the rate of intake does not change significantly across the session. Therefore, defining the parameters of the null distribution is an important decision. We believe this can be accomplished through comparison to (a) a water control group, if available, or (b) a uniform distribution of intake. Using a water group to parameterize this null distribution is optimal because rate of water intake in non‐deprived animals is typically constant throughout a session (Ardinger et al., 2020; Linsenbardt & Boehm, 2014, 2015). Therefore, if using this analysis approach, we would recommend using a water control group in initial experiments to allow direct comparisons in the rate of alcohol versus water intake as a function of access time. This approach has been used successfully in previous research. For example, DID is a common 2‐h alcohol access protocol which models binge drinking (Rhodes et al., 2005), giving animals single‐bottle access to 20% alcohol for 2 h a day, with water available the remaining 22 h. In studies using a water control group, between subjects' 20% alcohol and water groups (i.e., mice in the water group receive water for 2 h instead of alcohol) have the greatest divergence during the first 15 min of the DID session (Linsenbardt & Boehm, 2014), which is strong evidence for front‐loading during this time. Based upon these data, more recent studies have forgone the water control group and determined if alcohol front‐loading is present based upon this established 15‐min threshold (Ardinger et al., 2020, 2021). In these studies, we argued that since 15 min accounts for 12.5% of the total 2‐h DID session, rodents would need to consume significantly more (as determined using a one‐sample *t*‐test) than 12.5% of their total intake within the first 15 min of a DID session to be considered as having front‐loaded on a given day (i.e., 12.5% is the parameterized null distribution to compare to in this example). This sort of approach provides information regarding the skew of the drinking pattern. A simple comparison of drinking pattern to metabolic rate (as outlined above), is sufficient to provide evidence for intoxication.

Regardless of which analysis plan is chosen, researchers are encouraged to consider the most clinically and experimentally relevant definition of front‐loading when determining whether front‐loading occurred. Categorizations determined through comparison to a null distribution or through the outlined change point approach/provided code are only meant to serve as suggestions and potentially useful benchmarks.

## EXPERIENCE MATTERS: CHANGE IN FRONT‐LOADING ACROSS ALCOHOL ACCESS SESSIONS

Drugs are most rewarding when use results in rapid intoxication. For example, consider the difference between snorting cocaine versus chewing coca, both of which contain the same stimulant. Intranasal cocaine enters the bloodstream more quickly and at a higher concentration than cocaine from masticated coca leaves, which accounts for its greater rewarding effect and higher addiction potential (Grinspoon & Bakalar, 1981; Karch, 1999). The same rationale can be applied to front‐loading alcohol, where rapid consumption results in faster intoxication and, most often, greater reward. Alcohol front‐loading in many rodent models has been shown to increase progressively over days (Ardinger et al., 2020; Darevsky et al., 2019; Linsenbardt & Boehm, 2014, 2015; Rhodes et al., 2007; Salling et al., 2018; Wilcox et al., 2014), and has therefore been suggested to reflect a progressive increase in the motivation to experience alcohol's rewarding effects, or a pattern driven each session by an acute intensification of alcohol's intoxicating actions. Importantly, in studies which offer a water control group, there is no evidence of water front‐loading and/or change in water consumption patterns over days (Ardinger et al., 2020; Linsenbardt & Boehm, 2013, 2014; Rhodes et al., 2007). As animals repeatedly consume alcohol, they may learn that different rates of alcohol consumption yield differential subjective rewarding effects, a task akin to differential reinforcement of high rates of behavior (DRH) (Girolami et al., 2009). Further, studies have demonstrated that rodents with an alcohol drinking history will front‐load quinine‐adulterated alcohol (Bauer et al., 2021; Darevsky et al., 2019), and that mice with a water drinking history do not (Bauer et al., 2021). These findings support the idea that experience with alcohol consumption leads to an avidity for intoxication. This can be so strong that animals develop a “head down and push” strategy to consume this quinine adulterated alcohol, despite its aversive taste, to feel the rewarding effects of intoxication (Darevsky et al., 2019). This evidence suggests that following drinking experience, alcohol front‐loading is not exclusively driven by taste, which may be the case when animals exhibit intake patterns skewed toward the onset of access to sweetened solutions such as sucrose and saccharin (D'Aquila, 2010; Davis & Smith, 1992; Lardeux et al., 2013; Spector et al., 1998). Overall, consideration of when front‐loading initially emerges and if it escalates over days can offer information about the motivational factors influencing rapid alcohol consumption.

In addition to an increase in front‐loading over days, front‐loading has also been reported on the first day of alcohol access in a few alcohol‐naïve selectively bred high alcohol‐preferring mouse lines (Ardinger et al., 2020, 2021). These mice reliably consume pharmacologically relevant amounts of alcohol during both chronic and binge access models, and represent a rodent model of AUD (Oberlin et al., 2011). A high level of front‐loading in the first minutes of alcohol access in a naïve mouse may be indicative of a motivation to consume alcohol to experience its pre‐absorptive effects (i.e., taste or smell), as mice have not yet had a chance to experience the rewarding postabsorptive effects (driven by a pharmacologically relevant BEC) (Ardinger et al., 2020). Alternatively, or additionally, this observation may be influenced by alcohol's novelty on this first day of access. A relationship between novelty seeking and AUD risk is well‐described (Flagel et al., 2014; Manzo et al., 2014; for a review, see Wingo et al., 2016). In contrast, a drinking pattern in which front‐loading develops robustly and progressively over days may be indicative of developing motivation to consume alcohol quickly to experience the rewarding, intoxicating effects. For this reason, analysis of change in drinking patterns, including when front‐loading initially emerges and if/how it changes over days, and BECs to determine if subjects were intoxicated is critical in understanding the incentive value of alcohol and how it might be altered by alcohol drinking experience.

## FRONT‐LOADING CANNOT SOLELY BE DRIVEN BY METABOLIC TOLERANCE

An additional and/or alternative explanation for front‐loading which progressively increases over days of alcohol access (as described above) is that high initial intake is instrumental in overcoming developed chronic and/or metabolic tolerance—i.e., as days of alcohol access continue, more rapid alcohol consumption is required to feel the same rewarding effects. Chronic tolerance is a decrease in the effects of alcohol at a given dose following multiple, separate exposures (as opposed to acute tolerance, which is defined as a tolerance that occurs during a single session of intoxication; Kalant, 1998). Chronic tolerance may stem either from changes in the neural and behavioral sensitivity to alcohol, or an increase in the rate at which alcohol is metabolized, which is often referred to as metabolic tolerance. Both have been observed in high‐drinking rodent models after weeks of 24‐h, 2BC access (e.g., Matson et al., 2013 for metabolic tolerance; Matson et al., 2014 for chronic behavioral tolerance) as well as after repeated binge drinking experiences, which can lead to both chronic behavioral tolerance and metabolic tolerance; Linsenbardt et al., 2011. Regardless, alcohol drinking experience which engenders tolerance means that intake would have to be either more rapid or greater in quantity to achieve similar neural and psychological effects as in the naïve organism.

Considering metabolic tolerance first, as stated above, to reach a pharmacologically relevant blood EtOH concentration (BEC), individuals must consume alcohol at a higher rate than their liver metabolism, so increases in the rate of metabolism would, in principle, demand higher rates of drinking to achieve intoxication. However, the relationship between front‐loading and the development of metabolic tolerance is not clear. Assessment of metabolic tolerance (as determined by BEC 2‐h after an injection of 2 g/kg alcohol) in HAP2 and HAP3 mice (Ardinger et al., 2020) and C57BL/6J mice (Linsenbardt & Boehm, 2014) with a 2‐week binge drinking history showed no differences in post‐injection BEC between alcohol and water groups, suggesting that the front‐loading seen during the 2‐weeks of DID in the alcohol history group (and not the water history group) is not driven by differences in alcohol metabolism. On the contrary, using this same injection procedure, HAP1 mice (Linsenbardt & Boehm, 2015) with 2‐weeks of alcohol drinking history did display significantly lower BECs than water controls, suggesting some development of metabolic tolerance. Assessment of metabolic tolerance in C57BL/6J mice voluntarily consuming alcohol following forced alcohol exposure through the chronic intermittent EtOH (CIE) vapor procedure indicated no significant differences between slope of brain alcohol concentration in CIE or air (control) mice during the descending BEC limb, suggesting that the higher front‐loading seen in the CIE group was not driven by metabolic tolerance (Griffin et al., 2009). Further, if tolerance were the only factor which influences front‐loading, forced abstinence should decrease subsequent front‐loading, as is the case with total alcohol intake (O'Tousa & Grahame, 2016). Studies have demonstrated that this is not the case (Griffin et al., 2009; Robinson & McCool, 2015). Thus, although far from settled, the literature does not support a role for metabolic tolerance in the development of front‐loading over time.

Relationships between initial sensitivity during the ascending BEC limb and the development of behavioral tolerance have been reported (Khanna et al., 1985; Tabakoff et al., 1980). This is another reason that disentangling the contribution of tolerance and reward‐driven alcohol consumption may prove to be challenging. Within a single session, acute tolerance can be assessed through comparison of response to a given BEC on the ascending and descending limbs (Radlow, 1994, 2006). If acute (within a single session) tolerance has developed, an individual's response to a BEC on the descending limb will be lower than response to that same BEC on the prior ascending limb. This is an important factor in studying the development of AUD, as a blunted response to the negative, sedative effects felt during the descending BEC limb is predictive of the future development of AUD (King et al., 2014). To date, no studies have explicitly assessed if there is a relationship between front‐loading and acute tolerance. There is a renewed interest in the field to consider how various forms of tolerance contribute to the development of AUD (Elvig et al., 2021), and the inclusion of front‐loading analyses in future research will add value to this understanding.

As stated above, the current literature does not support a role for metabolic tolerance in front‐loading, and there is insufficient evidence to make a determination about the relationship between acute tolerance and front‐loading. Chronic behavioral tolerance, however, remains a candidate for increases in the rate of alcohol consumption over time. If alcohol intake during a session is affected by intoxication levels, then behavioral tolerance should either permit higher levels of intake without interfering with ingestive behavior or attenuate the sought‐out rewarding effects of alcohol intoxication leading to a compensatory increase in the rate of drinking. During chronic, 2BC alcohol access in selectively‐bred cHAP mice, escalation in voluntary intake is accompanied by increasing behavioral tolerance to alcohol's ataxic actions (Matson et al., 2014), while forced abstinence increased sensitivity to alcohol's ataxic effects as well as decreasing voluntary drinking (O'Tousa & Grahame, 2016), suggesting that this type of tolerance may drive increasing alcohol consumption. Unfortunately, these studies did not assess changes in drinking patterns, so it is not clear how these changes in alcohol sensitivity affected drinking patterns (as opposed to total daily intake), but it has been reported that cHAP mice front‐load during DID (Ardinger et al., 2021). Using the DID procedure, Linsenbardt et al. (2011) tested B6 mice for ataxia following a 2 h DID session. They found that alcohol consumption caused ataxia after 8 consecutive binge days, but not after 15 consecutive days, suggesting development of chronic tolerance. This was confirmed by measuring ataxia resulting from injected alcohol following 15 days of binge alcohol drinking or water only consumption. They found ataxic tolerance after 15 days of DID alcohol drinking. Although this study did not assess intake patterns, a later study used B6 mice and the DID procedure to show that front‐loading increased after 15 days of alcohol access, which suggests that front‐loading is driven, at least in part, by chronic tolerance (Linsenbardt & Boehm, 2014). Future studies could better assess how tolerance and front‐loading are related by manipulating the extent of tolerance and determining how this alters drinking patterns.

## FRONT‐LOADING: A ROLE FOR INCENTIVE SALIENCE?

Incentive salience describes a state of motivated behavior characterized by extreme “wanting” (Berridge et al., 1989; Berridge & Robinson, 2016), which may contribute to the development of alcohol use disorder (Cofresí et al., 2019; Olney et al., 2018; Robinson et al., 2013). Modifications of the ventral striatal dopamine system are critical in the expression of incentive salience. For example, increased dopamine in the posterior ventral tegmental area is associated with increased alcohol seeking (Hauser et al., 2011). Further, rats trained to self‐administer alcohol in an operant task display an increase in accumbal dopamine within the first 5 to 10 min of consumption, even though brain alcohol levels do not reach their peak until ~40 min into the session (Doyon et al., 2005). To the extent that dopamine release represents activity in the incentive salience system (Berridge, 2007; Robinson & Berridge, 1993), this work provides evidence that anticipation of intoxication may function as incentive salience prior to alcohol's direct, pharmacological actions in experienced subjects. Similarly, the frequency of drinking bouts in a given session has been described as a measure of incentive salience “wanting” (Robinson & McCool, 2015), and increased dopamine levels in dopamine transporter knock‐out (DAT KO) mice increases the frequency and duration of licking (Rossi & Yin, 2015).

Because front‐loading is characterized by early session intake, any front‐loading which occurs will directly contribute to BEC rise and stimulation. There is a growing body of literature which suggests that increased initial sensitivity to the acute rewarding effects of alcohol, such as greater reports of liking, wanting, and stimulant response, are predictive of subsequent heavy alcohol intake (Erblich & Earleywine, 2003; Holdstock et al., 2000; King et al., 2011, 2014; Newlin & Renton, 2010); reviewed in Ray et al. (2016) and de Wit and Phillips (2012). In rodent models, alcohol sensitivity/stimulation is regularly measured through the assessment of locomotor activity during intoxication, and prevailing theory suggests a relationship between drug‐stimulated locomotion and reward (Wise & Bozarth, 1987). Indeed, previous work indicates a strong, positive correlation between front‐loading during DID and concurrently‐measured, home‐cage locomotion (Linsenbardt & Boehm, 2015), further suggesting a relationship between the rewarding effect of alcohol and front‐loading, where mice who front‐load at a greater rate experience greater rewarding effects of alcohol. Also related may be recent work in humans allowed to intravenously self‐administer alcohol (within limits), showing that risky drinking patterns predict faster rates of self‐administration (Plawecki et al., 2016; Sloan et al., 2019). Together, this evidence suggests that there is a likely relationship between incentive salience and alcohol front‐loading.

There is also a relationship between incentive salience and context; reviewed in Valyear et al., 2017. In many of the front‐loading studies cited above (Table 1) alcohol drinking pattern is always assessed in the same context (typically a home‐cage or an operant box). When comparing levels of operant responding in a context with a conditioned stimulus (CS+) which indicates alcohol availability to a context where no alcohol is available, it has been demonstrated that the CS+ will elicit more alcohol seeking in the alcohol context (Millan et al., 2015; Remedios et al., 2014). Further, context plays a role in heavy drinking episodes (Stanesby et al., 2019). To our knowledge, no study has assessed if alcohol front‐loading changes depending on whether alcohol is associated with a given context. If incentive salience was a contributing factor in the maintenance of front‐loading, the hypothesis is that front‐loading would decrease in a context where alcohol has not previously been offered. However, we also note that an additional or alternative explanation for this finding could be that context‐dependent front‐loading is driven by context‐dependent tolerance, a phenomenon documented in alcohol use (González et al., 2019; White et al., 2002).

## FRONT‐LOADING: A DRINKING PATTERN DRIVEN BY NEGATIVE REINFORCEMENT?

Another additional and/or alternative explanation for the development of front‐loading is negative reinforcement. Negative reinforcement describes alcohol consumption specifically motivated by a desire to relieve anxiety, stress and/or withdrawal symptoms (Koob & Le Moal, 2008). The idea that negative reinforcement could reliably induce alcohol front‐loading directly competes with the current hypothesis, that front‐loading is driven by anticipation of alcohol's rewarding effects. There is an abundance of literature describing negative reinforcement to be driven by the brain's *antireward* system which does not begin to influence drug consumption until an individual displays drug dependence, for a review, see Koob and Le Moal (2008). This theory of addiction explains that drug reward is typically experienced in the earlier stages of the addiction cycle: preoccupation/anticipation and binge/intoxication, which is then thought to transition to the withdrawal/negative affect stage, where negative reinforcement is at its highest. The proposed mechanism of front‐loading is most aligned with the preoccupation/anticipation stage, where the hypothesis is that front‐loading is driven by an avidity for alcohol.

An alternative to the reward based hypothesis proposed herein is that negative reinforcement contributes to the development of alcohol front‐loading. Alcohol‐dependent rodents subsequently self‐administer more alcohol than non‐dependent controls (Becker & Lopez, 2004; Griffin et al., 2009; Lopez & Becker, 2005; O'Dell et al., 2004; Roberts et al., 2000; Robinson & McCool, 2015). Both higher front‐loading and total alcohol intake have been observed in subsequent voluntary alcohol consumption testing in mice exposed to alcohol vapor using a CIE vapor procedure as compared with air control; a well‐established model of alcohol dependence (Griffin et al., 2009; Robinson & McCool, 2015). However, in Griffin et al. (2009), the air control group *also* demonstrates front‐loading behavior, consuming around 63% of their total intake in the early part of the session (approximately 250 licks of 400 total consumed the first 40 min during the final testing session). These results indicate that CIE certainly exacerbates front‐loading but suggest that dependence is not necessary for front‐loading to develop; moreover, any history of vapor exposure would also greatly increase behavioral tolerance, another candidate for causing front‐loading (see above). In other words, vapor exposure studies cannot distinguish between tolerance and dependence as factors that would alter alcohol drinking patterns. In a clinical study, heavy drinkers were divided into “reward” and “relief/habit” groups based on their responses to the UCLA reward, relief, habit drinking scale (RRHDS; Grodin et al., 2019). One might expect greater negative reinforcement drinking in the relief/habit group. However, these groups did not differ in alcohol self‐administration behavior, although the pattern of responding during IV self‐administration was not examined in this study. Therefore, front‐loading may contribute to the higher alcohol intake seen in alcohol vapor‐exposed animals, and it is possible that negative reinforcement could be a driving factor in the maintenance of front‐loading behavior. However, this does not rule out the possibility that front‐loading is driven by alcohol's rewarding effects and it should also be noted that alcohol front‐loading has been reported in rodents who do not self‐administer alcohol at levels which would induce dependence (Bauer et al., 2021; Flores‐Bonilla et al., 2021; Jeanblanc et al., 2019; Linsenbardt & Boehm, 2014). Another way of summarizing this is that although negative reinforcement as a driver of front‐loading cannot be ruled out, limited current data suggest that it is neither necessary nor sufficient for front‐loading to develop. Current literature assessing alcohol front‐loading in the context of negative reinforcement and/or dependence is lacking. This is a major future direction and future research should aim to directly assess if there is a relationship between negative reinforcement and front‐loading.

## SEX DIFFERENCES IN FRONT‐LOADING?

Very few studies have assessed front‐loading in females and males. Note that much of the influential work discussed here only assessed intake patterns in male animals (Darevsky et al., 2019; Griffin et al., 2009; Linsenbardt & Boehm, 2014; Robinson & McCool, 2015; Salling et al., 2018; Wilcox et al., 2014). Female rodents typically outdrink males during alcohol self‐administration. This phenomenon has been reported across different species and self‐administration protocols (Li et al., 2019; Lourdes de la Torre et al., 2015; Oberlin et al., 2011; Priddy et al., 2017; Sneddon et al., 2020); and there is new evidence directly linking front‐loading in female rats as the reason for their higher total alcohol intake during operant self‐administration (Flores‐Bonilla et al., 2021). Further, it has been reported that female B6 mice with a binge drinking history front‐load more quinine‐adulterated alcohol than males (Bauer et al., 2021). Several key reviews have highlighted sex differences in AUD (Agabio et al., 2017; Becker & Koob, 2016; Flores‐Bonilla & Richardson, 2020; Verplaetse et al., 2021), however, few studies have assessed sex differences within front‐loading. There is growing evidence that human men and women are at similar risk for the development of AUD (Grant et al., 2017; White et al., 2015), and NIH now mandates the study of sex as a biological variable. Therefore, future careful consideration of sex differences in front‐loading will be critical in understanding the relationship between front‐loading and the development of AUD.

## FUTURE DIRECTIONS

In addition to the future directions described throughout the review (i.e., assessment of front‐loading in different contexts, further consideration of the relationship between negative reinforcement and front‐loading, and additional study of the association between acute tolerance and front‐loading), future research coupling behavioral evaluation with assessment of alcohol intake patterns will be necessary to further elucidate the relationship between motivation and front‐loading. Nonetheless, there is increasing evidence that front‐loading alcohol, at least in part, demonstrates motivation driven from alcohol's rewarding effects.

Future research may consider the relationship between “pre‐partying” or “pre‐gaming” and front‐loading. This well documented phenomenon is described as alcohol consumption prior to an event, see Foster and Ferguson (2014) for a review. While some literature uses “pre‐partying”, “pre‐gaming”, and “front‐loading” interchangeably (Borsari et al., 2016; Chaney et al., 2019; Wells et al., 2009; Yurasek et al., 2016), we note that the accepted definition of “pre‐partying/pre‐gaming” differs from the definition outlined for front‐loading in the current review as pre‐partying/pre‐gaming does not always mean a skew of alcohol consumption toward the onset of alcohol access. Indeed, the largest study examining pre‐partying indicates that individuals consume more drinks during the event than prior to it (Paschall & Saltz, 2007), suggesting there is not a skew associated with pre‐partying which we would consider to be front‐loading. It should be noted that Paschall and Saltz (2007) did not assess AUD symptoms, therefore, it is possible that individuals who regularly consumed alcohol prior to an event were more likely to develop AUD later in life. This phenomenon has been reported in previous research (LaBrie et al., 2016) and should be assessed in future work. Further, many pre‐partying studies present the number of days where pre‐partying occurred, but do not examine temporal intake pattern which would allow for determination of whether or not front‐loading occurred (LaBrie et al., 2011; Paves et al., 2012; Pedersen & LaBrie, 2007). However, given the clinical relevance of pre‐partying/pre‐gaming, future research may consider a larger focus on temporal intake within these pre‐partying/pre‐gaming studies.

Future work should also strive to elucidate the relationship between front‐loading and high intensity drinking (HID). Assessment of drinking patterns is incredibly relevant when studying high‐intensity drinking—a high‐risk pattern of alcohol consumption with recent, renewed interest in the field. HID can be defined as reaching a BEC twice or more of the NIAAA‐defined binge drinking threshold (Patrick & Azar, 2018). An entire body of literature exists which evaluates total alcohol intake using rodent models of AUD, (for a review, see Goltseker et al., 2019), but very few studies focus on rate of intake. Further assessment of drinking patterns will be needed to elucidate the relationship between HID and front‐loading.

In conclusion, front‐loading is an understudied drinking pattern which may represent a measure of motivation to consume alcohol, and directly relate to the future development of AUD. Further research is necessary to determine the role of avidity, tolerance, and negative reinforcement in front‐loading to unravel the contribution (if at all) of each construct to this intake pattern.

## CONFLICT OF INTEREST

The authors declare no conflict of interest.
